# Correction to ‘Buried treasure—marine turtles do not “disguise” or “camouflage” their nests but avoid them and create a decoy trail’

**DOI:** 10.1098/rsos.201031

**Published:** 2020-06-24

**Authors:** Thomas J. Burns, Rory R. Thomson, Rosemary A. McLaren, Jack Rawlinson, Euan McMillan, Hannah Davidson, Malcolm W. Kennedy

*R. Soc. Open Sci.* **7**, 200327 (Published online 13 May 2020) (doi:10.1098/rsos.200327)

This correction refers to an error in reference [12]. It should be replaced by the below:

**Reference**

12. McGowan A, Broderick AC, Deeming J, Godley BJ, Hancock EG. 2001 Dipteran infestation of loggerhead (*Caretta caretta*) and green (*Chelonia mydas*) sea turtle nests in northern Cyprus. *J. Nat. Hist.* **35**, 573–581. (doi:10.1080/00222930151098233)

This has now been corrected.

