# Supplementary material for: Buried treasure—marine turtles do not ‘disguise’ or ‘camouflage’ their nests but avoid them and create a decoy trail
Source: R Soc Open Sci. 2020 May 13;7(5):200327. doi: 10.1098/rsos.200327 (PMC7277256; doi:10.1098/rsos.200327)
Supplement: Sea turtle nesting decoy behaviour - Electronic supplementary material (ESM) [file RSOS200327supp1.pdf]

# Buried treasure - Marine turtles do not 'disguise' or 'camouflage' their nests but avoid them and create a decoy trail

Thomas J. Burns*, Rory R. Thomson, Rosemary A. McLaren, Jack Rawlinson, Euan McMillan, Hannah Davidson and Malcolm W. Kennedy

Institute of Biodiversity, Animal Health & Comparative Medicine, and School of Life Sciences, Graham Kerr Building, College of Medical, Veterinary and Life Sciences , University of Glasgow, Glasgow G12 8QQ, Scotland, UK.

*Present address - School of Life and Environmental Sciences, Centre for Integrative Ecology, Deakin University, Geelong (Burwood campus), VIC, 3216, Australia

Authors for correspondence: Malcolm Kennedy (malcolm.kennedy@glasgow.ac.uk) and Thomas Burns (tomb-09@hotmail.co.uk)

**Electronic supplementary material (ESM)**

**Appendix S1.** Discrimination of nesting behavioural phases.

**Table S1.** Comparison of candidate models used to examine factors influencing hawksbill turtle phase duration and respiration rates.

**Table S2.** Pairwise comparison of the duration of the excavation, oviposition (egg laying), refilling and sand-scattering behavioural phases for hawksbill turtles (*Eretmochelys imbricata*), averaged over four nesting seasons.

**Table S3.** Pairwise comparisons of the duration of each nesting phase of hawksbill turtles between and within each of four nesting seasons.

**Table S4.** The effect of curved carapace width and length on the duration of nesting phases of hawksbill turtles averaged over four nesting seasons.

**Table S5.** The effect of beach on the duration of nesting phases of hawksbill turtles averaged over four nesting seasons.

**Table S6.** Pairwise comparisons of breathing rates of hawksbill turtles between phases.

**Figure S1.** Sand-scattering movement maps of hawksbill turtles after refilling and covering the nest and before departure to the sea.

**Figure S2.** Comparison of durations of nesting phases from season to season for hawksbill turtles.

**Figure S3.** Sand-scattering movement maps of leatherback turtles after refilling and covering the nest and before departure to the sea.

**References** for electronic supplementary material

**Appendix S1.** Discrimination of nesting behavioural phases.

**Excavation.** Front flipper movements cease. Rear right and left flippers used alternately. Characterised by the flicking of sand to one side then the other, with movements gradually changing as the nest hole deepens.

**Oviposition.** Rear flipper actions cease. Leatherbacks leave one rear flipper partially inserted into the nest hole throughout oviposition, hawksbills place their rear flippers to each side of the nest hole. Egg laying begins and is accompanied by the periodic movement of the flipper muscles.

**Refilling.** Rear flipper movements begin with the lifting of the covering flipper from the entrance of the nest hole. The covering flipper is then generally used to push sand from within the sides of the nest hole on top of the eggs. Rear flippers are then used alternatively to push sand into the hole and press it downwards. Stronger compaction actions, where it is evident that significant weight is brought to bear occur with increasing frequency as the phase progresses.

**Sand scattering**. This phase is widely termed “camouflage” or “disguise” from the presumption that it functions to disguise the nest site against detection by predators or parasites. While this seems sensible, there is no evidence for its function so we have here used the neutral term “sand scattering”. Begins with the simultaneous movement of both front flippers (the first movement of them since the end of body pitting). The front flippers are moved in a powerful “butterfly stroke” fashion to throw sand backwards; this action is generally repeated several times during a movement event. Rear flippers are moved in a synchronised side-to-side “swishing” movement (similar to that seen during body pitting) that acts to scatter sand. Front and rear flipper movements do not occur at the same time but may occur in series within the same movement event. Changes in the position and orientation of the turtle on the beach and periods of complete cessation of all movement often associated with breathing also occur.

**Table S1.** Comparison of candidate models used to examine factors influencing hawksbill turtle nesting phase duration and respiration rates. The top five and ten candidate models shown for phase duration and respiration rate, respectively. CCW, curved carapace width; CCL, curved carapace length; df, degrees of freedom; AICc, second order Akaike Information Criterion.

| **Model** | **df** | **AICc** | **ΔAICc** | **Akaike weight** |
|---|---|---|---|---|
| **<u>Phase duration:</u>** | | | | |
| ~ phase + beach + year + CCW + CCL + phase:beach + phase:year + phase:CCW + phase:CCL | 33 | 10604 | 0 | 1.00 |
| ~ phase + beach + year + CCW + CCL + phase:beach + phase:year + phase:CCW | 30 | 10619.1 | 15.16 | 0.00 |
| ~ phase + beach + year + CCW + phase:beach + phase:year + phase:CCW | 29 | 10619.9 | 15.87 | 0.00 |
| ~ phase + beach + year + CCW + CCL + phase:beach + phase:year + phase:CCL | 30 | 10624.7 | 20.67 | 0.00 |
| ~ phase + beach + year + CCL + phase:beach + phase:year + phase:CCL | 29 | 10628.9 | 24.9 | 0.00 |
| **<u>Respiration rate:</u>** | | | | |
| ~ phase | 9 | 849.9 | 0 | 0.56 |
| ~ phase + beach | 10 | 852 | 2.02 | 0.20 |
| ~ phase + year | 10 | 852.7 | 2.74 | 0.14 |
| ~ phase + beach + year | 11 | 854.7 | 4.79 | 0.05 |
| ~ phase + CCW | 10 | 857.6 | 7.62 | 0.01 |
| ~ phase + beach + phase:beach | 13 | 858 | 8.06 | 0.01 |
| ~ phase + beach + CCW | 11 | 859.5 | 9.55 | 0.01 |
| ~ phase + year + phase:year | 13 | 859.8 | 9.82 | 0.00 |
| ~ phase + year + CCW | 11 | 860.3 | 10.36 | 0.00 |
| ~ phase + beach + year + phase:beach | 14 | 860.8 | 10.9 | 0.00 |

**Table S2. .** Pairwise comparison of the duration of the excavation, oviposition (egg laying), refilling and sand-scattering behavioural phases for hawksbill turtles (*Eretmochelys imbricata*), averaged over four nesting seasons (2014, 2015, 2016 and 2017). Comparisons computed with a Tukey adjustment (emmeans; emmeans package, ref. (1)), from the phase fixed effect in the final selected linear mixed effects model (lme; nlme package, ref. (2)), which included behavioural phase of nesting, year, beach, curved carapce width, curved carapace length and two-way interactions between phase and each of the former, and individual as a random effect. SE, standard error; and CI; 95% confidence intervals; *, confidence intervals that do not cross zero. Degrees of freedom = 575. With regard to phase duration variation between years and influence of carapace dimensions, the duration of each nesting phase did not vary significantly across years (see exception for excavation in this table), but the relative duration of phases differed across years; for instance, strong contrasts between phases apparent in 2015 were not found in other years (figure S2; table S3).

| Contrast | Estimate | SE | Lower CI | Upper CI |
|---|---|---|---|---|
| refilling - excavation | -321 | 64.6 | **-487.4*** | **-154*** |
| refilling - oviposition | -465 | 37.1 | **-560.1*** | **-369*** |
| refilling - sand-scattering | -218 | 39.9 | **-320.5*** | **-115*** |
| excavation - oviposition | -144 | 68.6 | -320.5 | 33 |
| excavation - sand-scattering | 103 | 70.2 | -77.8 | 284 |
| oviposition - sand-scattering | 247 | 46.1 | **128.1*** | **366*** |

**Table S3.** Pairwise comparisons of the duration of each nesting phase of hawksbill turtles between and within each of four nesting seasons. Comparisons computed with a Tukey adjustment (emmeans; emmeans package), from the phase-year interaction in the final selected linear mixed effects model (lme; nlme package), which included behavioural phase of nesting, year, beach, curved carapce width, curved carapace length and two-way interactions between phase and each of the former, and individual as a random effect. SE, standard error; and CI; 95% confidence intervals; *, confidence intervals that do not cross zero. Degrees of freedom = 575.

*Table follows on next page.*

| Parameter | Contrast | | | | | |
|---|---|---|---|---|---|---|
| **Phase** | **Year 1** | **Year 2** | **Estimate** | **SE** | **Lower CI** | **Upper CI** |
| excavation | 2014 | 2015 | -19.28 | 159.6 | -568.57 | 530 |
| | 2014 | 2017 | 343.82 | 158.5 | -201.78 | 889.4 |
| | 2014 | 2018 | 492.24 | 203.7 | -208.93 | 1193.4 |
| | 2015 | 2017 | 363.1 | 95.7 | **33.73*** | **692.5*** |
| | 2015 | 2018 | 511.52 | 166.8 | -62.67 | 1085.7 |
| | 2017 | 2018 | 148.42 | 164.5 | -417.76 | 714.6 |
| oviposition | 2014 | 2015 | -107.03 | 70.6 | -350.08 | 136 |
| | 2014 | 2017 | 81.76 | 74.9 | -176.08 | 339.6 |
| | 2014 | 2018 | -44.38 | 98.9 | -384.71 | 296 |
| | 2015 | 2017 | 188.79 | 64.5 | -33.11 | 410.7 |
| | 2015 | 2018 | 62.66 | 92 | -254.08 | 379.4 |
| | 2017 | 2018 | -126.13 | 92.4 | -443.97 | 191.7 |
| refilling | 2014 | 2015 | 76.93 | 45.7 | -80.4 | 234.3 |
| | 2014 | 2017 | 121.86 | 49 | -46.78 | 290.5 |
| | 2014 | 2018 | 189.33 | 67.5 | -42.99 | 421.7 |
| | 2015 | 2017 | 44.92 | 44.8 | -109.38 | 199.2 |
| | 2015 | 2018 | 112.4 | 64.9 | -110.92 | 335.7 |
| | 2017 | 2018 | 67.48 | 66.4 | -161.18 | 296.1 |
| sand-scattering | 2014 | 2015 | -52.14 | 71.9 | -299.7 | 195.4 |
| | 2014 | 2017 | 30.12 | 79 | -241.89 | 302.1 |
| | 2014 | 2018 | -30.6 | 106.3 | -396.37 | 335.2 |
| | 2015 | 2017 | 82.27 | 71.8 | -164.72 | 329.2 |
| | 2015 | 2018 | 21.54 | 102.5 | -331.08 | 374.2 |
| | 2017 | 2018 | -60.73 | 105.7 | -424.64 | 303.2 |
| **Year** | **Phase 1** | **Phase 2** | **Estimate** | **SE** | **Lower CI** | **Upper CI** |
| 2014 | refilling | excavation | -428.02 | 151.2 | -948.34 | 92.3 |
| | refilling | oviposition | -350.17 | 69 | **-587.67*** | **-112.7*** |
| | refilling | sand-scattering | -107.48 | 68.7 | -343.87 | 128.9 |
| | excavation | oviposition | 77.84 | 158.3 | -466.85 | 622.5 |
| | excavation | sand-scattering | 320.54 | 158.3 | -224.25 | 865.3 |
| | oviposition | sand-scattering | 242.7 | 83.5 | -44.75 | 530.1 |
| 2015 | refilling | excavation | -524.23 | 73.4 | **-777*** | **-271.5*** |
| | refilling | oviposition | -534.14 | 50.7 | **-708.52*** | **-359.8*** |
| | refilling | sand-scattering | -236.55 | 53 | **-419*** | **-54.1*** |
| | excavation | oviposition | -9.91 | 80.1 | -285.58 | 265.8 |
| | excavation | sand-scattering | 287.68 | 81.7 | **6.5*** | **568.9*** |
| | oviposition | sand-scattering | 297.59 | 62 | **84.11*** | **511.1*** |
| 2017 | refilling | excavation | -206.06 | 74.9 | -463.98 | 51.9 |
| | refilling | oviposition | -390.27 | 57.2 | **-587.2*** | **-193.4*** |
| | refilling | sand-scattering | -199.21 | 63.7 | -418.48 | 20.1 |
| | excavation | oviposition | -184.22 | 82.1 | -466.67 | 98.2 |
| | excavation | sand-scattering | 6.85 | 86.8 | -291.78 | 305.5 |
| | oviposition | sand-scattering | 191.07 | 72 | -56.78 | 438.9 |
| 2018 | refilling | excavation | -125.11 | 160.2 | -676.5 | 426.3 |
| | refilling | oviposition | -583.88 | 95.2 | **-911.37*** | **-256.4*** |
| | refilling | sand-scattering | -327.41 | 104.8 | -687.96 | 33.1 |
| | excavation | oviposition | -458.77 | 169.8 | -1043.19 | 125.7 |
| | excavation | sand-scattering | -202.3 | 175.5 | -806.24 | 401.6 |
| | oviposition | sand-scattering | 256.47 | 119 | -153.04 | 666 |

**Table S4.** The effect of curved carapace width and length on the duration of nesting phases of hawksbill turtles averaged over four nesting seasons (2014, 2015, 2016 and 2017). Comparisons computed with a Tukey adjustment (emtrends; emmeans package), from the final selected linear mixed effects model (lme; nlme package), which included behavioural phase of nesting, year, beach, curved carapce width, curved carapace length and two-way interactions between phase and each of the former, and individual as a random effect. Our results also suggest that curved carapace width affects the excavation phase, with turtles with larger widths excavating their nest holes more quickly. SE, standard error; df, degrees of freedom; CI; 95% confidence intervals; and *, confidence intervals that do not cross zero. Degrees of freedom = 575.

| Phase | Trend | SE | Lower CI | Upper CI |
|---|---|---|---|---|
| **Curved carapace width** | | | | |
| excavation | -31.021 | 12.46 | **-55.5*** | **-6.539*** |
| oviposition | -0.725 | 6.54 | -13.57 | 12.118 |
| refilling | -8.297 | 4.42 | -16.99 | 0.393 |
| sand-scattering | 6.784 | 6.93 | -6.83 | 20.399 |
| **Curved carapace length** | | | | |
| excavation | 7.25 | 6.09 | -4.714 | 19.21 |
| oviposition | 6.29 | 3.28 | -0.155 | 12.73 |
| refilling | -2.68 | 2.19 | -6.975 | 1.62 |
| sand-scattering | -1.5 | 3.48 | -8.346 | 5.34 |

**Table S5.** The effect of beach on the duration of nesting phases of hawksbill turtles averaged over four nesting seasons (2014, 2015, 2016 and 2017). Comparisons computed with a Tukey adjustment (emmeans; emmeans package), from the phase-beach interaction in the final selected linear mixed effects model (lme; nlme package), which included behavioural phase of nesting, year, beach, curved carapce width, curved carapace length and two-way interactions between phase and each of the former, and individual as a random effect. SE, standard error; df, degrees of freedom; and CI; 95% confidence intervals. Degrees of freedom = 575.

| Phase; Campbleton-Hermitage contrast | Estimate | SE | Lower CI | Upper CI |
|---|---|---|---|---|
| excavation | -161.64 | 95.3 | -451.48 | 128.2 |
| oviposition | 84.88 | 61.2 | -101.29 | 271.05 |
| refilling | 81.15 | 42.8 | -48.96 | 211.26 |
| sand-scattering | 28.67 | 68 | -178.29 | 235.64 |

**Table S6.** Pairwise comparisons of breathing rates of hawksbill turtles between phases. Comparisons computed with a Tukey adjustment (emmeans; emmeans package), from the phase fixed effect in the final selected linear mixed effects model (lme; nlme package), which included behavioural phase of nesting and individual as a random effect. SE, standard error; df, degrees of freedom; and CI; 95% confidence intervals. Degrees of freedom = 212.

| Contrast | Estimate | SE | Lower CI | Upper CI |
|---|---|---|---|---|
| refilling - excavation | 0.1335 | 0.208 | -0.404 | 0.671 |
| refilling - oviposition | 0.9236 | 0.147 | 0.543* | 1.304* |
| refilling - sand-scattering | 0.2013 | 0.157 | -0.206 | 0.608 |
| excavation - oviposition | 0.7901 | 0.182 | 0.32* | 1.26* |
| excavation - sand-scattering | 0.0678 | 0.191 | -0.426 | 0.562 |
| oviposition - sand-scattering | -0.7223 | 0.122 | -1.037* | -0.407* |

**Figure S1.** Sand-scattering movement maps of hawksbill turtles after refilling and covering the nest and before departure to the sea. A representative sample of ten out of 28 track maps. The position of the nest is indicated by a red-filled circle and nest site as indicated 'NS', the sand-scattering stations by circles. For distances between stations, angles of turn for individuals whose identification numbers are given, see supplementary data file 'Hawksbill nesting data decoy Burns et al'.  From the 2018 season. Maps re-sized to fit and scale bars included. All original maps recorded are available from the corresponding authors.  Turtle silhouette not to scale.

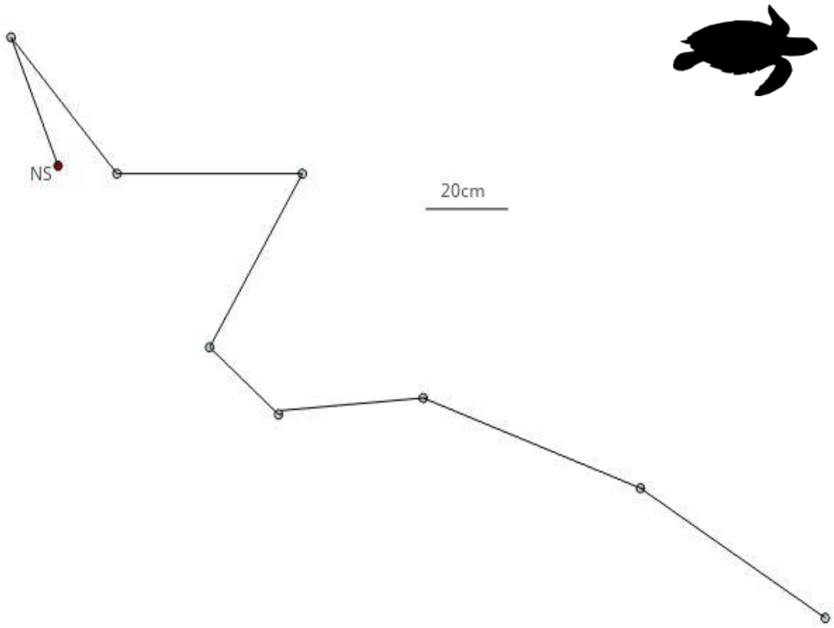


3

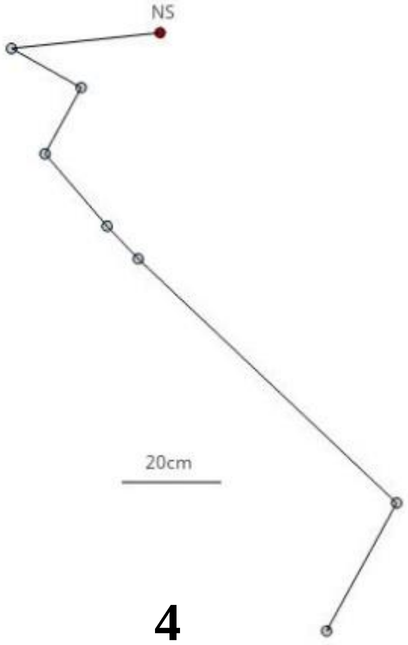


4

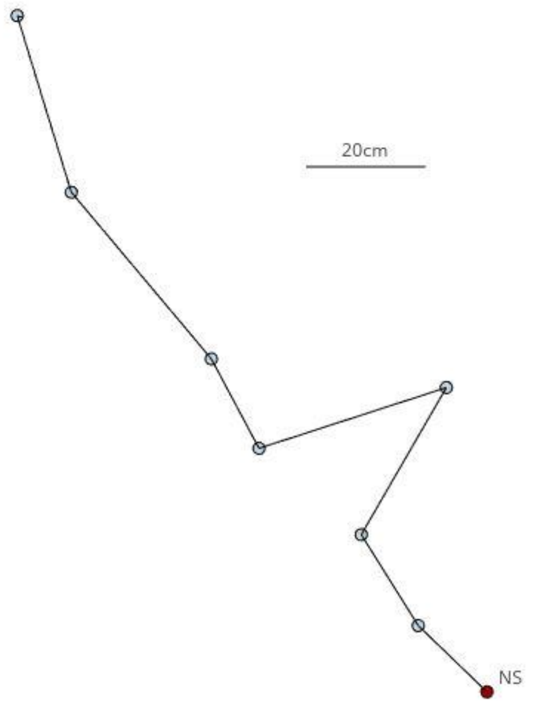


**7**

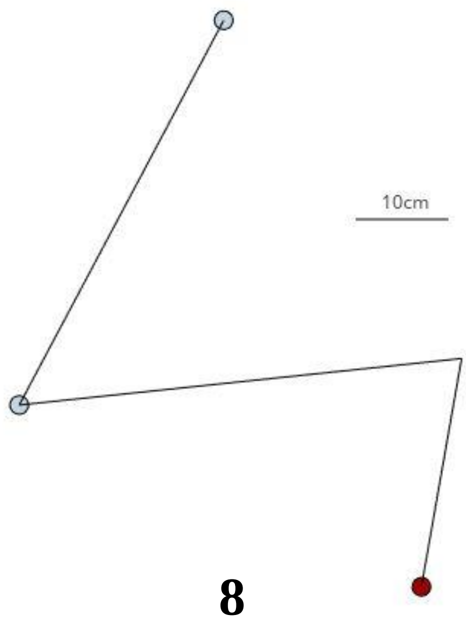


**8**

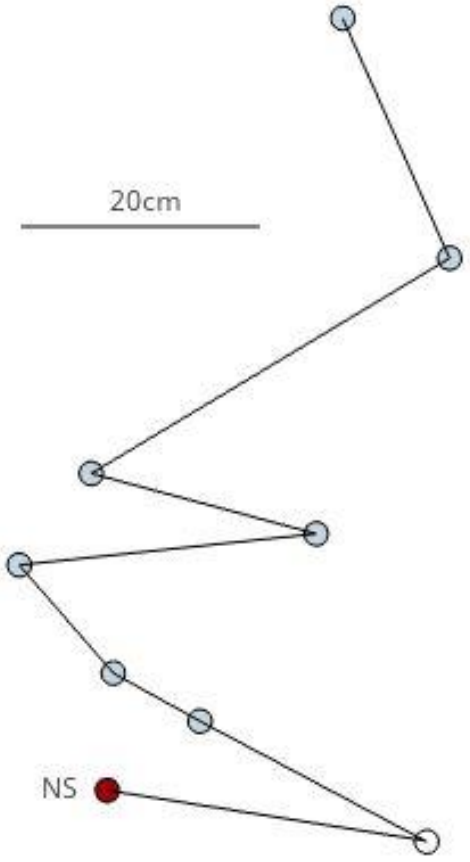


**19**

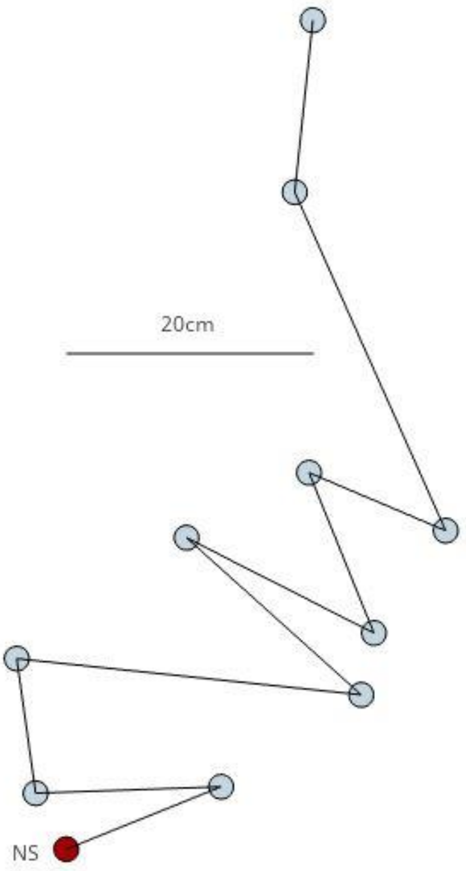


**20**

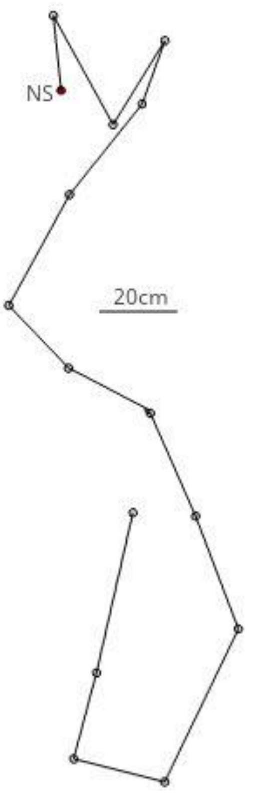


**15**

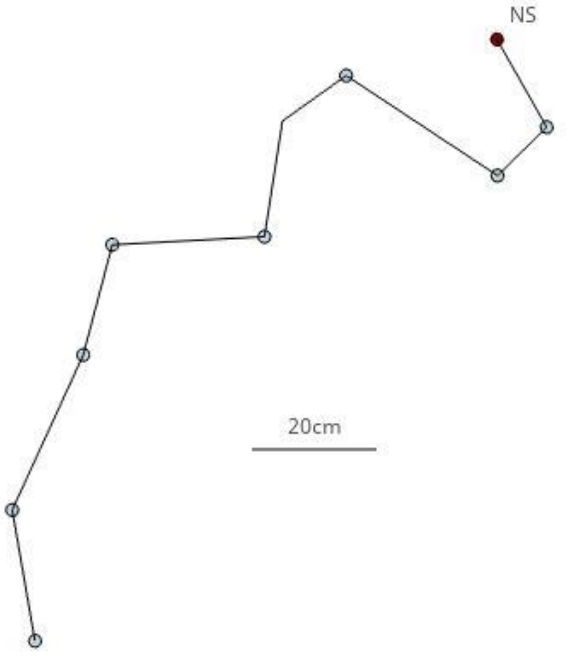


**16**

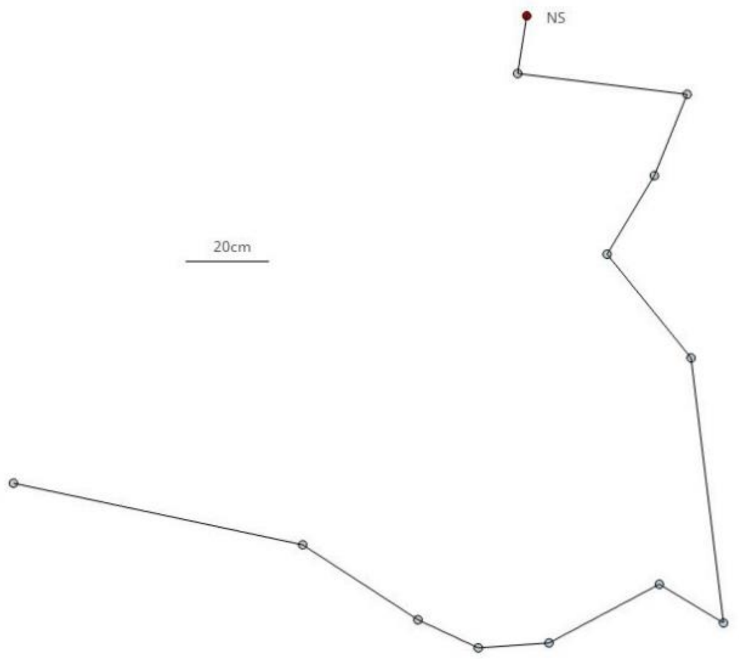


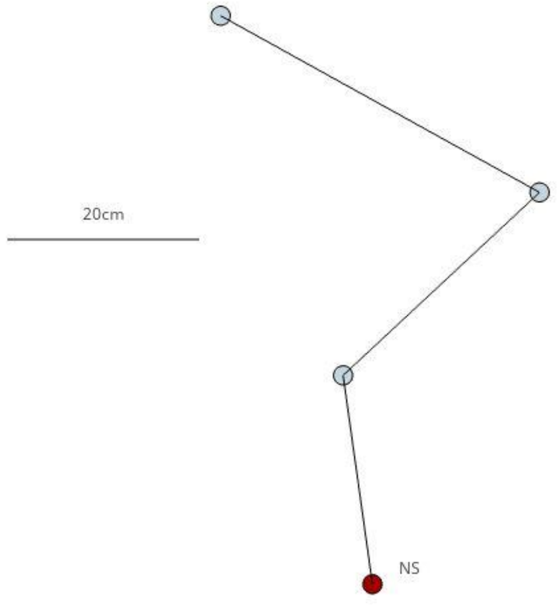


**17**

**18**

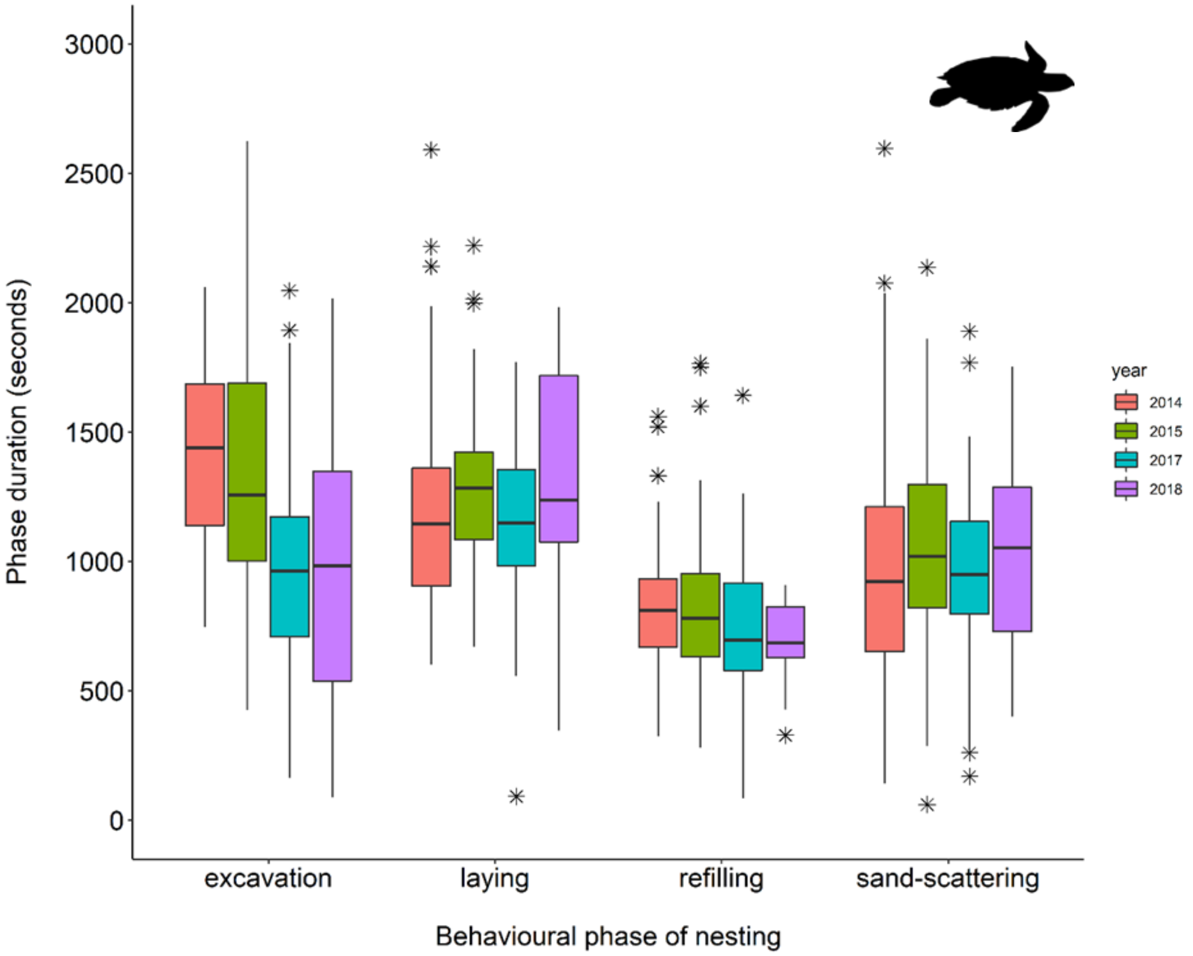


**Figure S2.** Comparison of durations of nesting phases from season to season for hawksbill turtles. Boxes show 25 and 75 % quartiles, central line the median, whisker bars show the largest and smallest values within 1.5 * interquartile range below and above the 25$^{th}$ and 75$^{th}$ percentiles, respectively, and * represents outliers.

**Figure S3.** Sand-scattering movement maps of leatherback turtles after refilling and covering the nest and before departure to the sea. A representative sample of ten out of 42 track maps recorded in 2019 and redrawn to scale. The position of the nest is labelled, the orientation of the turtle while oviposition is indicated by the dashed line, with the arrow showing the direction turtle was facing. Sand scattering stations are indicated by the filled circles. Maps re-sized to fit page and individual scale bar included for each. For distances between stations, angles of turn for individuals whose identification numbers (ID #) are given, see supplementary data file 'Leatherback nesting data decoy Burns et al'.  All original 2019 maps recorded from the field are available from the corresponding authors. For track maps from 2014 see ref. (3). Turtle silhouette not to scale.

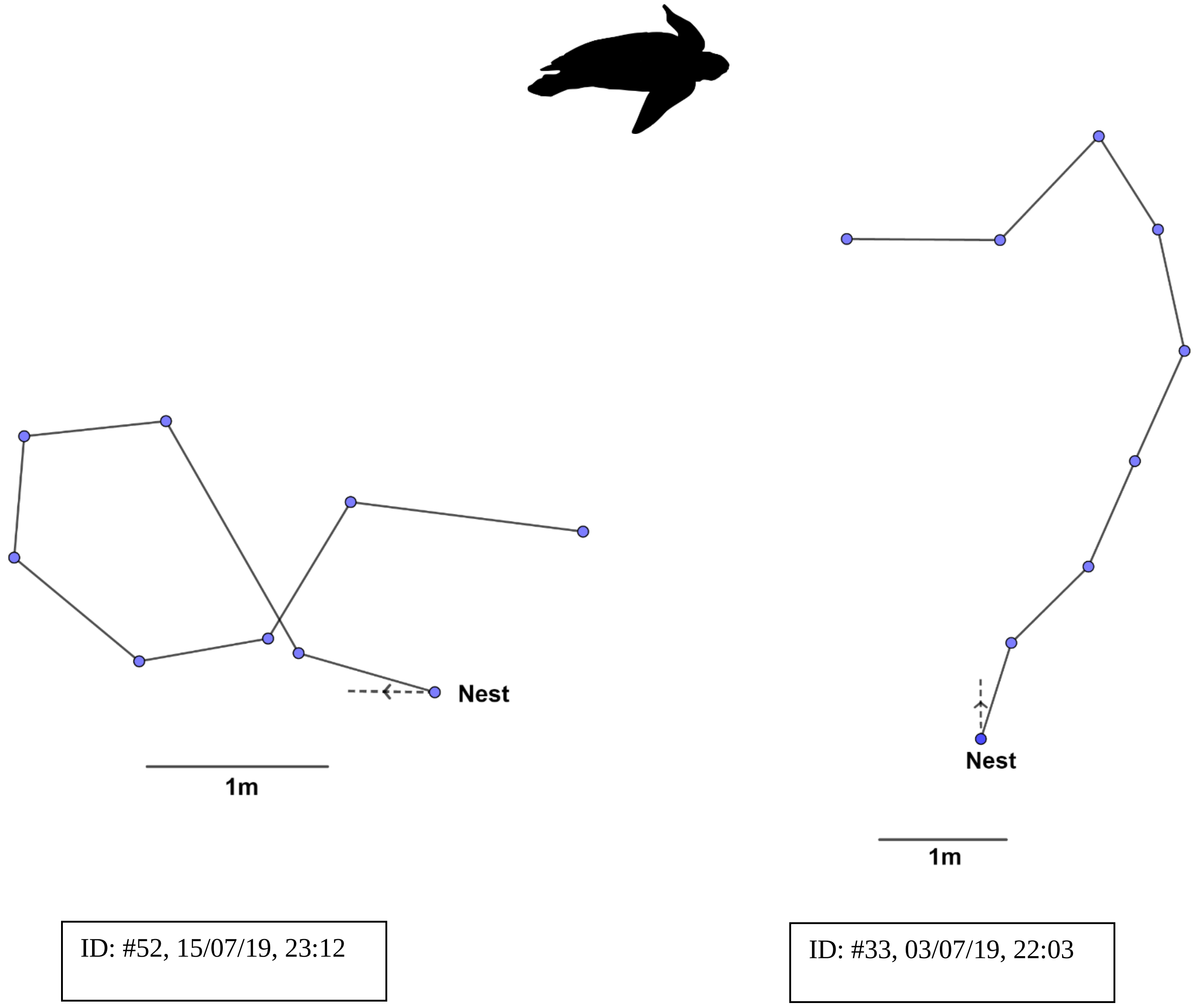

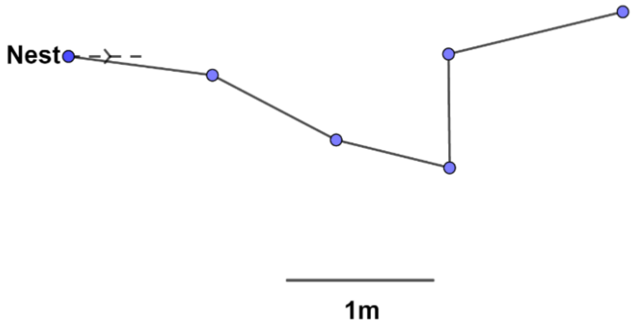


ID: #54, 15/07/19, 00:05

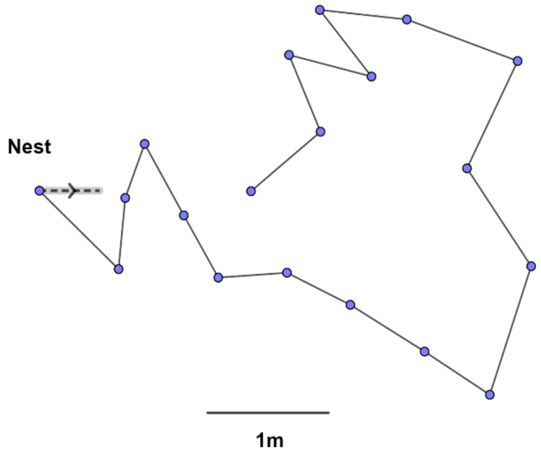


ID: #18, 25/06/19, 21:11

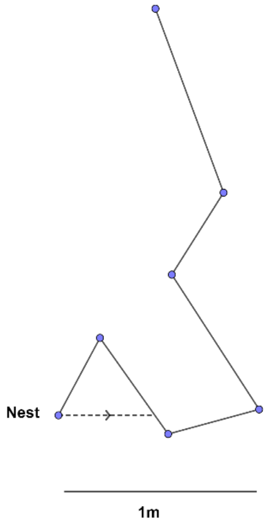


ID: #31, 31/07/19, 23:30

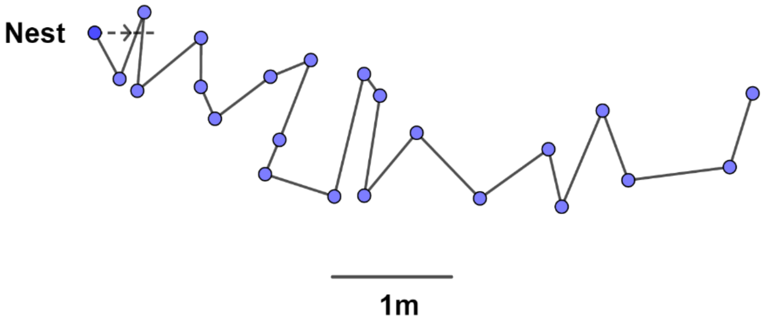


ID: #12, 21/06/19, 21:57

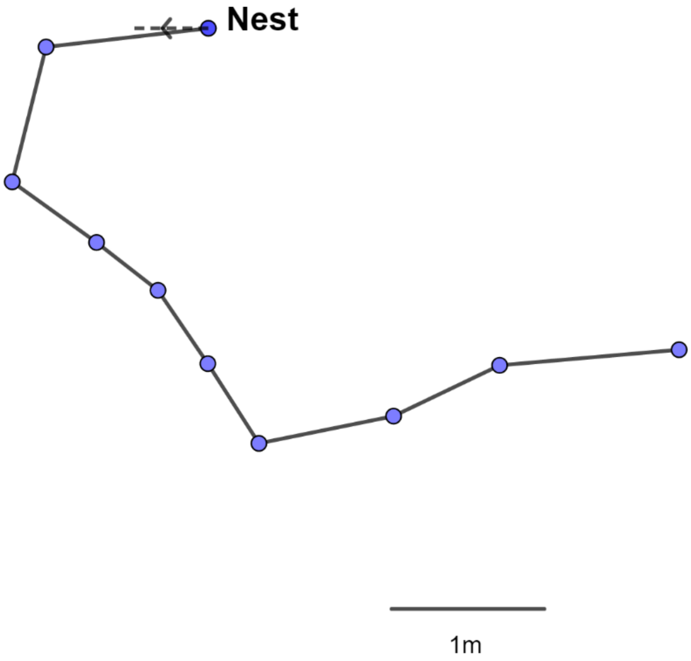


ID: #16, 23/06/19, 21:20

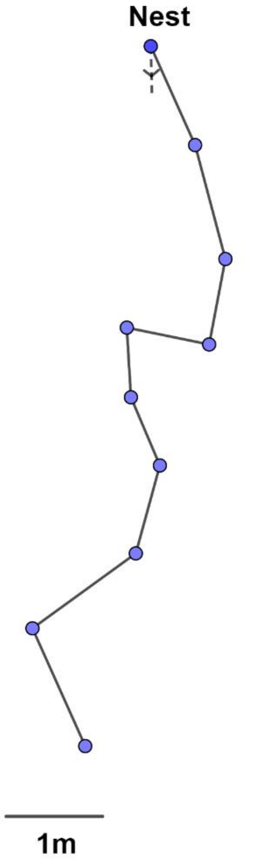


ID: #59, 20/07/19, 22:05

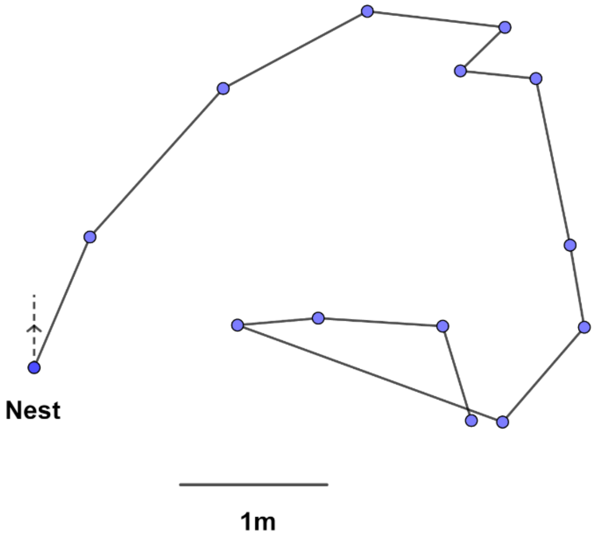


ID: #14, 21/06/19, 00:09

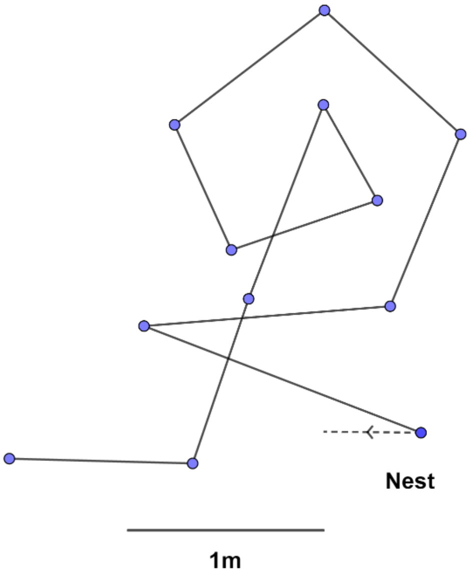


ID: #60, 20/07/19, 00:21

## References for electronic supplementary material


1. Lenth RV. emmeans: Estimated Marginal Means, aka Least-Squares Means. R package version 1.4.2. 2019 [Available from: https://CRAN.R-project.org/package=emmeans.
2. Pinheiro J, Bates D, DebRoy S, Sarkar D. _nlme: Linear and Nonlinear Mixed Effects Models_. R package version 3.1-1372018. Available from: https://CRAN.R-project.org/package=nlme.
3. Burns TJ, Davidson H, Kennedy MW. Large-scale investment in the excavation and "camouflaging" phases by nesting Leatherback Turtles (*Dermochelys coriacea*). Canadian Journal of Zoology. 2016;94(6):443-8.
